# Activated microglia nibbling glycosaminoglycans from spinal cord perineural nets: a new mechanism for neuropathic pain

**DOI:** 10.1038/s41392-022-01162-0

**Published:** 2022-09-22

**Authors:** Christa E. Müller, Tobias Claff

**Affiliations:** PharmaCenter Bonn and Pharmaceutical Institute, Pharmaceutical & Medicinal Chemistry, University of Bonn, An der Immenburg 4, 53121 Bonn, Germany

**Keywords:** Molecular neuroscience, Inflammation

**Fig. 1:**
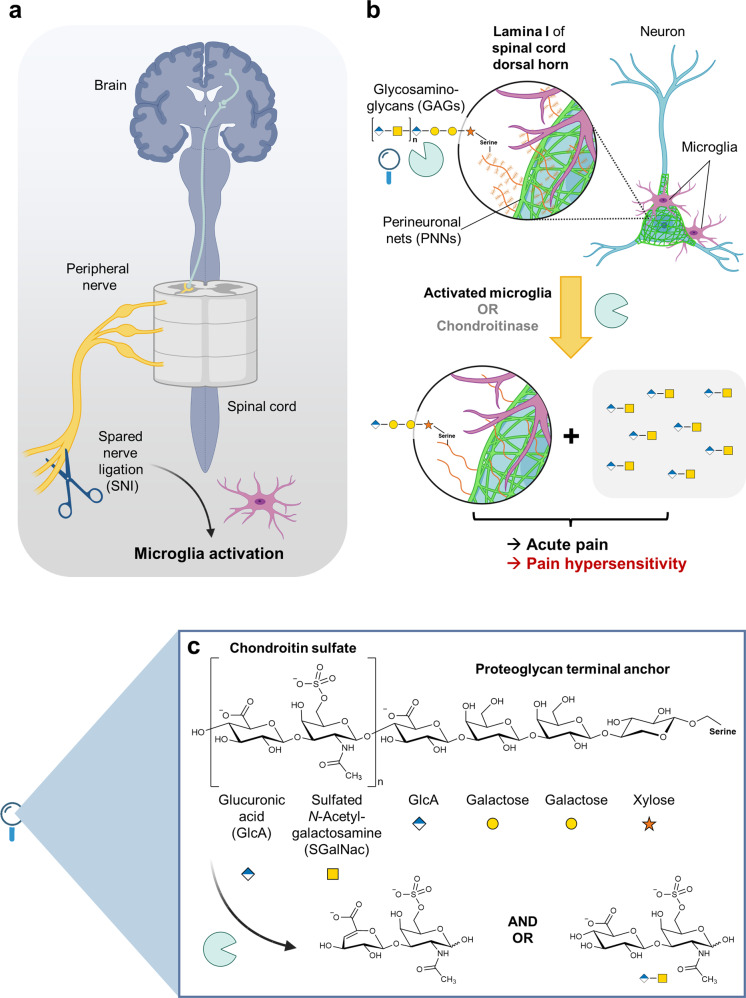
**a** Development of neuropathic pain by peripheral nerve injury, which leads to microglia activation in the spinal cord. **b** Activated microglia degrade perineural nets (PNNs) of nerves in the spinal cord dorsal horn lamina I leading to acute pain and the development of long-lasting pain hypersensitivity (allodynia). This effect of microglia can be imitated by chondroitinase treatment. In the present study, chondroitinase ABC (systematic name: chondroitin ABC lyase) was employed, a bacterial enzyme from *Proteus vulgaris*, that degrades polysaccharides such as chondroitin 4-sulfate and chondroitin-6-sulfate producing disaccharides. It cleaves off the side chains of chrondroitin sulfate proteoglycans. Treatment with this enzyme has previously been proposed for the treatment of spinal injuries. Chondroitin sulfate proteoglycans are glycoproteins to which chondroitin sulfate side chains are attached. They are secreted from cells and have various functions. For example, they inhibit axon regeneration after spinal cord injury. **c** Glycosaminoglycans (GAGs, also termed mucopolysaccharides) are linear, acidic polysaccharides that are composed of disaccharide units, an uronic acid (mostly glucuronic acid) which forms a 1,3-glycoside linkage with an amino sugar (e.g. *N*-acetylglucosamine). Chondroitin sulfate is a sulfated polysaccharide consisting of disaccharide units of D-glucuronic acid (or <10% L-iduronic acid) and *N*-acetylgalactosamine. It contains about 1 sulfate per disaccharide. The figure has been created with BioRender.com

In a recent study published in *Science*,^1^ Tansley, Khoutorsky et al. unraveled a novel mechanism by which activated microglia can induce pain and pain hypersensitivity. It involves the degradation of perineural nets (PNNs) around neurons in the spinal cord dorsal horn lamina I (Fig. 1).

PNNs are extracellular matrix structures present around specific neuronal cell bodies and nearby neurites, found in the central nervous system, e.g. in the brain cortex as well as in the spinal cord; they are often associated with inhibitory interneurons. PNNs are composed of proteoglycans that are decorated with glycosaminoglycans (GAGs) consisting mainly of chondroitin sulfate, termed chondroitin sulfate proteoglycans.^2^ Thus, PNNs are negatively charged macromolecules (for structure, see Fig. 1).

Peripheral nerve injury can lead to allodynia, a long-lasting hypersensitivity, e.g. to mechanical or thermal stimuli that normally do not cause pain. Neuropathic pain associated with allodynia and hyperalgesia, i.e. strongly increased sensitivity to pain stimuli, has been estimated to affect up to 10% of the population.^3^ This type of chronic pain is difficult to treat with current analgesic drugs. Thus, there is a huge medical need to develop novel, efficient therapies for neuropathic pain. Elucidating the underlying pathophysiology is a crucial prerequisite for the subsequent identification of novel drug targets.

Here, Tansley, Khoutorsky et al. performed mouse studies applying a spared nerve injury (SNI) model of allodynia. In this model, two of the three branches of the sciatic nerve are ligated resulting in mechanical (and thermal) hypersensitivity in the paw, with a maximal pain response peaking two to three days after surgery and lasting for at least 14 days; the other paw serves as a control. Upon peripheral nerve injury, microglia in the spinal cord are activated. For the first time, the authors additionally observed a decrease in GAGs (detected with *Wisteria floribunda* agglutinin that binds selectively to GAG sugar side chains present in chondroitin sulfate proteoglycans and thus in PNNs), while the PNN core protein was still detectable. Three days after the injury, when the pain response was maximal, GAGs could be detected in the lysosomes of microglia indicating that microglia had split off the GAGs from the PNNs and subjected them to lysosomal degradation.

The authors showed that genetically induced depletion of microglia abolished the development of pain hypersensitivity. Moreover, in the absence of microglia, the GAGs were not cut off from the PNNs around lamina I projection neurons.

Another line of evidence confirmed the importance of microglia for the development of pain hypersensitivity in the applied animal model. The chemokine receptor CX3CR1 is known to be involved in microglia activation and stimulation of phagocytotic activity.^4^ CX3CR1 knockout (KO) mice were subjected to the SNI model. They showed microgliosis comparable to wildtype animals, but had a decreased number of microglial lysosomes indicating reduced phagocytotic activity, and they developed no pain hypersensitivity. This indicates that the phagocytotic activity of microglia was essential for the development of allodynia. Moreover, no PNN degradation, and reduced lysosomal GAG accumulation was observed in these KO mice.

Subsequently, PNNs around lamina I were genetically disrupted, leading to spontaneous pain and thermal pain hypersensitivity. Using a complementary approach, GAGs were selectively removed from lamina I of PNNs by expression of the bacterial enzyme chondroitinase ABC (Fig. 1). This led to spontaneous pain and pain hypersensitivity in the absence of microglial activation.

To study the effects on a cellular level, patch-clamp recordings using spinal cord lamina I preparations surrounded by PNNs were performed ex vivo. The disruption of PNNs after peripheral nerve injury decreased inhibitory inputs form lamina I PNN-positive projection neurons, an effect that was dependent on microglia.

Since recent reports suggested sex differences in the role of microglia with respect to the development of neuropathic pain,^5^ Tansley, Khoutorsky et al. performed studies in male and female mice. However, no differences were observed.^1^

Previous studies investigating the role of activated microglia in neuropathic pain often focused on signaling by released molecules, e.g. adenosine triphosphate (ATP) or cytokines. This is the first study showing an (unexpected) enzymatic activity due to microglia activation resulting in the release of sulfoglycosides from PNNs, which are extracellular matrix structures consisting of chrondroitin sulfate proteoglycans, eventually inducing pain hypersensitivity. It remains unclear, how the discovered degradation of PNNs and GAGs leads to the observed changes in neuronal signaling and the induction of pain and allodynia. The described mechanism requires and warrants further study. It might eventually contribute to the discovery of novel targets and drugs for the treatment of neuropathic pain.
